# Helminth burden and ecological factors associated with alterations in wild host gastrointestinal microbiota

**DOI:** 10.1038/ismej.2016.153

**Published:** 2016-12-16

**Authors:** Lindsay K Newbold, Sarah J Burthe, Anna E Oliver, Hyun S Gweon, Christopher J Barnes, Francis Daunt, Christopher J van der Gast

**Affiliations:** 1 NERC Centre for Ecology & Hydrology, Wallingford, UK; 2 NERC Centre for Ecology & Hydrology, Penicuik, UK; 3 National History Museum of Denmark, University of Copenhagen, Copenhagen, Denmark; 4 Manchester Metropolitan University, School of Healthcare Science, Manchester, UK

## Abstract

Infection by gastrointestinal helminths of humans, livestock and wild animals is common, but the impact of such endoparasites on wild hosts and their gut microbiota represents an important overlooked component of population dynamics. Wild host gut microbiota and endoparasites occupy the same physical niche spaces with both affecting host nutrition and health. However, associations between the two are poorly understood. Here we used the commonly parasitized European shag (*Phalacrocorax aristotelis*) as a model wild host. Forty live adults from the same colony were sampled. Endoscopy was employed to quantify helminth infection *in situ*. Microbiota from the significantly distinct proventriculus (site of infection), cloacal and faecal gastrointestinal tract microbiomes were characterised using 16S rRNA gene-targeted high-throughput sequencing. We found increasingly strong associations between helminth infection and microbiota composition progressing away from the site of infection, observing a pronounced dysbiosis in microbiota when samples were partitioned into high- and low-burden groups. We posit this dysbiosis is predominately explained by helminths inducing an anti-inflammatory environment in the proventriculus, diverting host immune responses away from themselves. This study, within live wild animals, provides a vital foundation to better understand the mechanisms that underpin the three-way relationship between helminths, microbiota and hosts.

## Introduction

Helminth (including, nematodes, cestodes and trematodes) infections are common in humans, livestock and wild animal populations, having key ecological and evolutionary roles through the costs they impose on their hosts (Sheldon and Verhulst, 1996). There is clear evidence that high burdens of helminths are associated with increased morbidity in humans and significantly reduced production levels in livestock (Bethony *et al.*, 2006). However, impacts on free-living wild animal hosts are less understood, in particular the chronic and sublethal effects of infections (Tompkins *et al.*, 2011). Parasites may impact their hosts by directly competing for resources and, through immunoregulation, by altering trade-offs in investment between immunity and other core activities such as growth and reproduction (Maizels *et al.*, 2004). Immunoregulation by helminths is a mechanism that includes, suppression, diversion or conversion of host immune responses to the benefit of the parasite (Maizels *et al.*, 2004).

An additional component that coexists with parasites, and impacts how resources are utilised by the host, is the gastrointestinal microbiota. Parasites and microbiota can potentially interact within the gut, and such associations have the potential to affect host health as well as the microbiota and helminth populations themselves (Glendinning *et al.*, 2014). However, analogous to research in host–parasite interactions, studies of host–microbiota interactions have predominately focused on human and livestock hosts, establishing that the microorganisms composing the animal gut microbiota co-evolve with their hosts and have important functional roles in metabolism, nutrition and immunity (Glendinning *et al.*, 2014).

Few studies have investigated interactions between gut microbiota and parasites in hosts, with those that have focusing on intestinal helminths associated with significant morbidity in human infection, via impacts on growth and nutrition (Bethony *et al.*, 2006). In particular, whipworm (*Trichuris* spp.) and roundworm (*Ascaris* spp.) infections and interactions with faecal microbiota of infected human and murine model hosts have recently been reported (for example, Hayes *et al.*, 2010; Cooper *et al.*, 2013; Lee *et al.*, 2014; Houlden *et al.*, 2015; Kay *et al.*, 2015). Interestingly, one study proposed that helminth infection shifts the composition of gut microbiota, through promoting outgrowth or suppression of bacterial taxa, which consequently help regulate host immunity (Walk *et al.*, 2010).

Current understanding of how endoparasite infection impacts on wild animal hosts has yet to recognise or incorporate the role of host microbiota, which are a significant component of diversity within hosts, creating much of the biotic environment in which parasites interact (Rynkiewicz *et al.*, 2015). To our knowledge, only one study has considered associations between parasites and microbiota in wild hosts; which, using euthanized wild mice, found the relative abundances of certain microbial taxa co-varied with helminths colonizing the gut of that host (Kreisinger *et al.*, 2015).

Key reasons for this lack of understanding are host–microbiota studies have been predominately human focused, and studies of host–parasite systems have previously been impeded by difficulties associated with detecting and measuring endoparasite burdens in wild hosts (Burthe *et al.*, 2013). On the latter point, faecal egg counts or necropsy are often the only available methods for quantifying individual parasite burdens. However, destructive sampling, as in the Kresinger *et al.* (2015) study, is not an option if the host is of conservation importance and precludes investigation of sublethal effects and temporal patterns in infection that require longitudinal data. Faecal egg counts can be an unreliable method, with several limitations, including: density-dependent worm fecundity, temporal variation in egg shedding rates, lack of egg shedding by larval parasite stages, poor sensitivity at low worm burdens, and difficulty in sampling faeces of wild hosts in the field (Burthe *et al.*, 2013). Endoscopy is a promising new method for quantifying endoparasite infection in wild hosts, facilitating direct measurements of parasites *in situ* (Burthe *et al.*, 2013). This method was found to be a rapid, reliable and repeatable method for investigating individual variation and temporal changes in endoparasite burden within European shags (*Phalacrocorax aristotelis*).

Here we investigate associations between helminth infection burden and gut microbiota in a seabird host. European shags (hereafter shags) are commonly infected with the anisakid nematode, *Contracaecum rudolphii*, which infects the upper gastrointestinal tract, specifically the proventriculus and lower oesophagus (Farjallah *et al.*, 2008; Rokicki *et al.*, 2011; Burthe *et al.*, 2013). As the definitive host, shags become infected with third stage larvae via their fish diet, which develop into sexually mature adults that attach to the mucosal lining of the proventriculus (true stomach) and lower oesophagus (Burthe *et al.*, 2013). Although their effects are usually sublethal, these parasites compete with the host for nutrients, can cause pathological damage, and trigger costly immune responses (Hoberg, 2005; Fagerholm and Overstreet, 2009). In shags, infection has been shown to impair growth and survival of offspring (Reed *et al.*, 2008; Granroth-Wilding, 2014, 2015). In the current study, we extend this work to use shags as a model wild host–parasite–microbiota system. It is now recognized that there are discrete habitats and distinct microbiota compositions through the gastrointestinal tract (Donaldson *et al.*, 2016). Consequently, 40 breeding adult shags were sampled, with bacterial microbiota from three connected gastrointestinal microbiomes (including the proventriculus (site of infection), cloaca and faeces) assessed using 16S rRNA gene-targeted high-throughput sequencing, and had parasite burdens quantified *in situ* using endoscopy. Here we employed an approach from metacommunity theory that has proved useful in understanding host-associated microbiota, the partitioning of local constituent taxa which comprise a metacommunity, into core and satellite taxa (Magurran and Henderson, 2003). The core group consists of dominant taxa that are widely distributed and highly abundant across samples, contributing significantly to ecosystem function (Fuhrman, 2009, Magurran and Henderson, 2011), and the satellite group taxa are typically rare, occurring in low abundance at a limited number of samples, representing a seedbank of diversity (Prosser *et al.*, 2007; van der Gast *et al.*, 2011). Objectively partitioning these common and rare groups from a spatial or temporal metacommunity reveals important aspects of species abundance distributions which would otherwise be neglected without such a distinction (van der Gast *et al.*, 2011; Hedin *et al.*, 2015).

Combined, this facilitated one of the first assessments of microbiota–helminth associations in live wild animal hosts through a detailed examination of: (1) how the local communities were structured and to what extent stochastic and deterministic assembly regulated the core and satellite microbiota within each microbiome metacommunity; (2) how burdens of the nematode, *C*. *rudolphii,* were associated with each of the gastrointestinal microbiomes; and (3) whether such associations were only localised at the site of infection or if downstream effects through these microbiomes occurred.

## Materials and methods

### Study and sampling

Fieldwork was undertaken between 20 and 25 June 2013 on the Isle of May National Nature Reserve, Scotland (56°11′N, 2°33′W) as part of the Isle of May Long-Term Study (IMLOTS) of seabird populations, part of the Centre for Ecology and Hydrology's (CEH) network of long-term monitoring sites for detecting effects of environmental and climate change. As part of the IMLOTS population study, the laying date and breeding success (number of chicks fledged) of study individual was recorded from frequent nest checks using standardized protocols (Burthe *et al.*, 2013). Individuals were sexed from voice, size and behaviour. Individuals were caught during the early chick-rearing period and their body mass measured to the nearest 10 *g*. Endoscopy was performed under UK Home Office Project Licence PPL60/4001 and conducted by trained personnel (SJB) holding a personal license (PIL40/6722). The work had full ethical approval from the CEH Ethics Committee and the UK Home Office. Endoscopy of conscious adult shags was undertaken, as previously described (Burthe *et al.*, 2013), with full details provided in Supplementary Materials.

Gastrointestinal microbiota samples, across the 40 adult shags sampled, were taken when endoscopy was performed for each individual bird. Cloacal (*n*=39) and faecal (*n*=10) samples were collected directly with sterile viscose tipped swabs (Scientific Laboratory Supplies Ltd, Wilford, UK). A lower number of faecal samples were obtained as adult shags rarely defecate during the procedure. In addition, samples were obtained from the mucosal lining of the proventriculus (*n*=38) by swabbing the recessed camera lens of the withdrawn gastroscope, see Supplementary Materials. All swabs were stored at −20 °C immediately after sampling, and later transferred to a −80 °C freezer before DNA extraction.

### DNA extraction and sequencing

Gut microbiota DNA was extracted from sample swabs using the PowerSoil-htp 96 Well Soil DNA Isolation Kit, (Mobio Laboratories Inc., Carlsbad, CA, USA). Briefly, swab tips were excised into PowerSoil-htp bead plates containing PowerSoil-htp bead solution and solution C1. These plates were incubated at 60 °C for 20 min, then horizontally vortexed for a further 20 min at 2000 r.p.m. Following these additional lysis steps manufacturers recommended protocol was followed. Approximately 20–30 ng of template DNA was amplified using Q5 high-fidelity DNA polymerase (New England Biolabs, Hitchin, UK) each with a unique dual-index barcode primer combination (Kozich *et al.*, 2013). Individual PCR reactions employed 25 cycles of an initial 30 s, 98 °C denaturation step, followed by annealing phase for 30 s at 53 °C, and final extension step lasting 90 s at 72 °C. Primers were based upon the universal primer sequence 341F and 806R. An amplicon library consisting of ~550 bp amplicons spanning the V3-V4 hypervariable regions of the 16S rRNA gene, was sequenced at a concentration of 5.4 pm with a 0.6 pm addition on an Illumina MiSeq platform using V3 chemistry (Illumina Inc., San Diego, CA, USA). Details are given in the Supplementary Material.

### Sequence analysis

Sequenced paired-end reads were joined using PEAR (Zhang *et al.,* 2014), quality filtered using FASTX tools (Hannon, http://hannonlab.cshl.edu) and chimeras were identified and removed with ChimeraSlayer (Haas *et al.*, 2011). The sequences were clustered into operational taxonomic units with UCLUST (Edgar, 2010) as part of the QIIME package (Caporaso *et al.*, 2010) and representative sequences were selected (pick_rep_set.py, QIIME). The taxonomy of representatives was determined by QIIME's UCLUST consensus taxonomy assigner (assign_taxonomy.py, QIIME) using the Greengenes database release 13_2 (McDonald *et al.*, 2012). Resultant operational taxonomic units were combined to create phylotypes, associated at the 97% identity similarity cut-off, which roughly corresponds to a species/genus level (Tindall *et al.*, 2010). As an additional measure the identity of reference sequences from the most abundant operational taxonomic unit within each phylotype was corroborated using phylogenetic association. To control for putative kit contaminants process negative control samples, were included in analyses and potential false positives accounted for (Salter *et al.*, 2014). Taxonomic affiliation of each phylotype was further corroborated through use of phylogenetic association (Supplementary Figure S1), full details provided in Supplementary Materials. The raw sequence data reported in this study have been deposited in the European Nucleotide Archive under study accession number PRJEB10889. The relevant barcode information for each sample is shown in Supplementary Table S1.

### Statistical analysis

Taxa were partitioned into core and satellite microbiota groups as previously described (van der Gast *et al.*, 2011). Fisher's alpha index of diversity, a measure that is relatively unaffected by variation in sample size, and completely independent if *N* individuals>1000 (Magurran, 2004), within each local community sample was performed using PAST v3.01 (http://folk.uio.no/ohammer/past). Two-sample *t*-tests, regression analysis, coefficients of determination (*r*^2^), residuals and significance (*P*) were calculated using XLSTAT (v2015.1.01, Addinsoft, Paris, France). To test, to what extent, microbiota assembly within each microbiome was driven by stochastic or deterministic niche considerations, local communities were compared using a Monte Carlo procedure (1000 randomizations) to determine whether any two communities with each microbiome were more or less similar than would be expected by chance using the Raup and Crick probability-based index of similarity (van der Gast *et al.*, 2008). The Raup and Crick index, Bray–Curtis quantitative index of dissimilarity, analysis of similarities (ANOSIM) and similarity of percentages (SIMPER) were performed using PAST. Bray–Curtis index was used as the underpinning community dissimilarity measure for both ANOSIM and SIMPER. Canonical correspondence analysis (CCA) was used to relate the variability in the distribution of microbiota samples within microbiomes to host variables. Variables that significantly explained variation in the microbiota were determined with forward selection (1000 Monte Carlo permutations; *P*<0.05) and used in CCA (Hazard *et al.*, 2013). CCA was performed in CANOCO v5 (ter Braak and Smilauer, 2012). Volcano plots were generated using the differential expression package within XLSTAT and helminth burdens categorized using a modification of a previously described burden categorization (Burthe *et al.*, 2013). Specifically, helminth counts from individual hosts were categorized into high and low-burden groups; the high group was greater than the median helminth count of 21 (range 22–45 helminths) and the low group was equal to or less than the median count (range 3–21).

## Results

Intrinsic variables for the individuals sampled are summarised in Table 1. Across the variables measured, sex and mass (g) were significantly correlated (two-sample *t*-test, *t*=6.32, *P*<0.0001) with male birds (mean=1896.2 g±121.9 g s.d. of the mean) heavier than females (1614.0 g±103.1 g). In addition, host sex and nematode burden were correlated with males having significantly higher (*t*=2.17, *P*=0.037) nematode counts (*n*=28±14.8) than females (*n*=20±8.1).

Microbiota samples from the proventriculus (*n*=38), cloaca (*n*=39) and faeces (*n*=10) were collected from across the forty adult shags with diversity and microbiota composition assessed using targeted 16S rRNA gene-targeted high-throughput sequencing. From the 87 samples, a total of 6 332 244 bacterial sequence reads were included in the final analyses, identifying 350 genera and 487 distinct phylotypes classified to genus/species level using phylogenetic affiliation (Supplementary Figure S1); however, given the relative length of the ribosomal sequences analysed, these identities should be considered putative. The average numbers of bacterial sequence reads per sample were similar among the three microbiomes: proventriculus, 71 658±50 856; cloaca, 75 474±52 037; and faeces, 66 575±31 625. The relative abundance and distribution of these phylotypes was analysed within a metacommunity framework.

**Figure 1:**
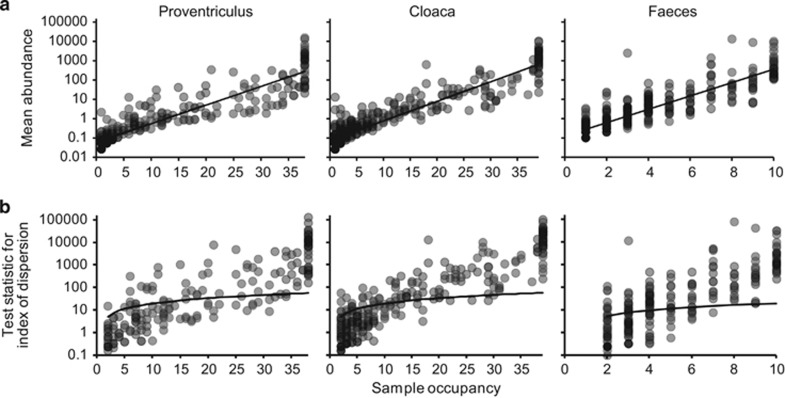
Distribution and dispersal of bacterial taxa among the proventriculus, cloacal and faecal microbiomes. (**a**) The number of samples for which each detected bacterial taxon was observed, plotted against the mean sequence abundance (log_10_ scale) of that taxon among all samples within each microbiome (proventriculus, *r*^2^=0.75, *F*_1,289_=855.5, *P*<0.0001; cloaca, *r*^2^=0.82, *F*_1,407_=1797.6, *P*<0.0001; and faeces, *r*^2^=0.71, *F*_1,332_=780.1, *P*<0.0001). (**b**) A dispersal plot to identify which bacterial taxa are randomly distributed within each microbiome, a measure used to assign core versus satellite status. Index of dispersion was calculated as the ratio of variance to mean of abundance for each taxon within each cohort and plotted for each sample. The line depicts the 2.5% confidence limit for the *χ*^2^ distribution. Taxa that fall below this line are randomly distributed and were considered satellite taxa, whereas those that are above the line are non-randomly distributed and were considered core taxa. The 97.5% confidence limit was not plotted, as no taxa fell below that line.

A coherent metacommunity would be expected to exhibit a significant positive distribution-abundance relationship (Guo *et al.*, 2000). Consistent with this prediction, for each microbiome, the abundance of individual bacterial phylotypes were significantly correlated with the number of local communities (samples) they were found to be present in Figure 1a. Therefore, as has been observed in traditional ecological studies of animal and plant species, the commonness and rarity of bacterial taxa in the proventriculus, cloacal and faecal metacommunities were found to be related to their permanence in the constituent local communities (Magurran and Henderson, 2003). Distribution-abundance relationships were objectively partitioned into core and satellite microbiota groups by decomposing the overall distribution using the ratio of variance to the mean abundance for each taxon. The variance to mean ratio, or index of dispersion, is an index used to model whether taxa follow a Poisson distribution, falling between the 2.5 and 97.5% confidence limits of the *χ*^2^ distribution (Krebs, 1999). The indices of dispersion were plotted against local community occupancy for taxa in each microbiome metacommunity (Figure 1b). Out of the 291 taxa that comprised the proventriculus metacommunity, 126 taxa were randomly distributed in space, that is, those taxa that fell below the 2.5% confidence limit line. Taxa that occurred only in a single sample were excluded from this analysis, as their dispersion in space would have no variance. For the purposes of the current study, those 69 taxa with the 126 randomly distributed taxa were classified as satellite taxa and the remaining 96 non-randomly distributed taxa classified as core microbiota group members. Out of the 409 taxa in the cloacal metacommunity, 141 were core and 268 formed the satellite group (including 103 taxa found in single samples only). Out of the 334 taxa in the faecal metacommunity, 139 were core and 195 satellite taxa (98 single sample taxa). Further, core group microbiota accounted for the majority or relative abundance in each microbiome: proventriculus, 99.9% cloaca, 99.9% and faeces, 99.8%.

**Figure 2:**
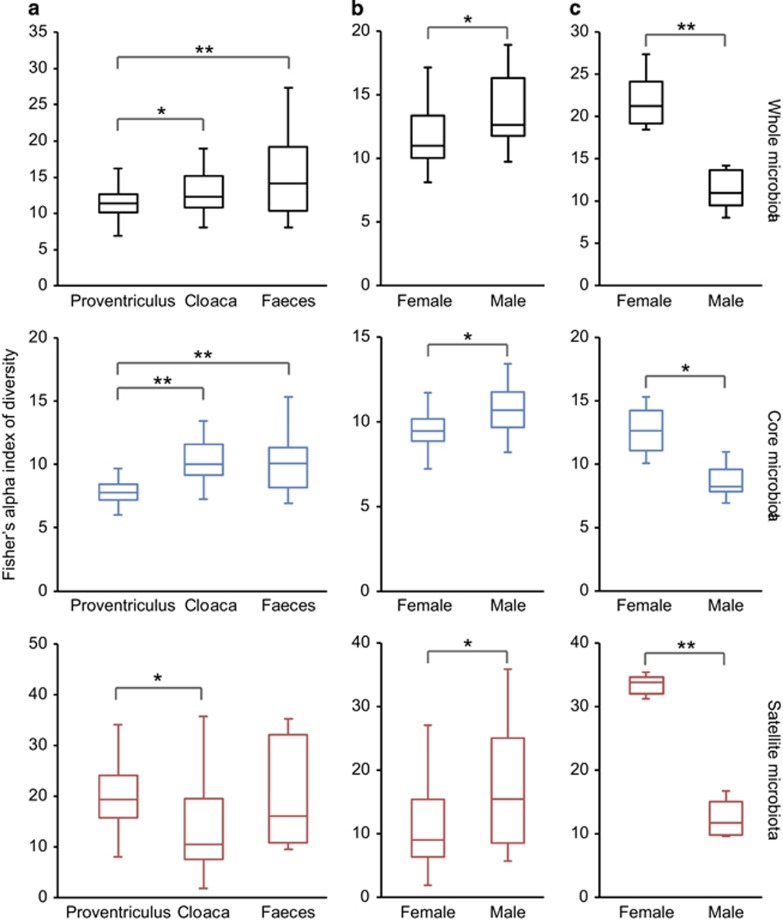
Box-plot comparisons of bacterial diversity, using Fisher's alpha index of diversity, of the whole, core and satellite microbiota (**a**) between microbiomes, and by host sex within the (**b**) cloacal and (**c**) faecal microbiomes. The top and bottom boundaries of each box indicate the 75th and 25th quartile values, respectively, and lines within each box represent the 50th quartile (median) values. Ends of whiskers mark the lowest and highest diversity values in each instance. Asterisks denote significant differences in comparisons of diversity at the *P*<0.05 level determined by two-sample *t*-tests (*P<0.05 and **P<0.005).

Bacterial diversity between microbiomes was compared using Fisher's alpha index of diversity. Mean local community diversity was significantly lower in the proventriculus when compared with the cloacal (two-sample *t*-test, *t*=2.37, *P*=0.01) and faecal microbiota (*t*=3.33, *P*<0.001) (Figure 2a); the cloacal and faecal microbiota were not significantly different. These patterns of diversity were also reflected between the core microbiota groups (*t*=8.07, *P*<0.0001 and *t*=4.52, *P*<0.0001, respectively) (Figure 2a). Between satellite microbiota groups, only the proventriculus and cloaca were significantly different (*t*=2.42, *P*=0.01), where the latter had lower diversity (Figure 2a). Tests for associations between diversity and intrinsic variables for each microbiome revealed that cloacal diversity was significantly correlated with host sex (Figure 2b). For the whole (*t*=2.28, *P*=0.036), core (*t*=2.43, *P*=0.02) and satellite (*t*=2.17, *P*=0.044) microbiota diversity was significantly higher in males than in females. Conversely, diversity was significantly higher in females than males for the faecal whole (*t*=5.58, *P*<0.001), core (*t*=3.29, *P*=0.011) and satellite (*t*=10.31, *P*<0.0001) microbiota (Figure 2c). No other significant associations between diversity and intrinsic variables were detected.

**Figure 3:**
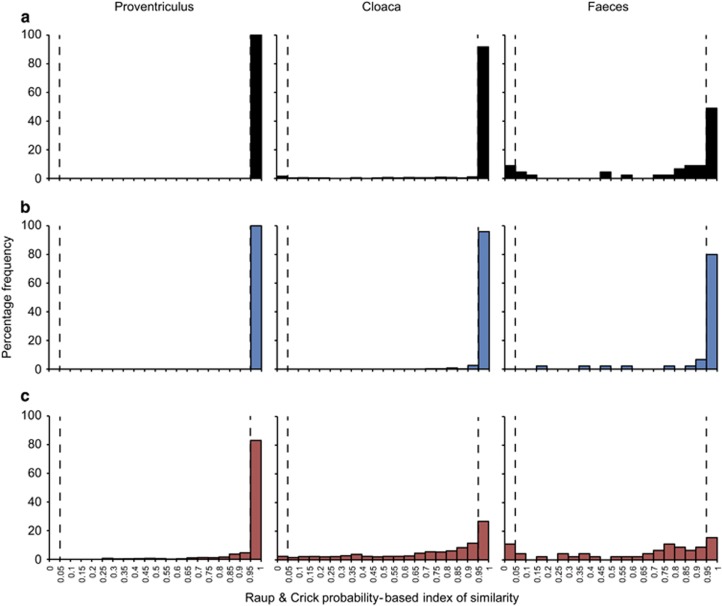
Distribution of pair-wise Raup and Crick (*S*_RC_) probability-based index similarity frequencies for the (**a**) whole, (**b**) core and (**c**) satellite microbiota within each microbiome. *S*_RC_>0.95 indicate microbiota significantly less different than expected by chance. *S*_RC_<0.05, microbiota significantly more different from each other than by chance. *S*_RC_>0.05 and *S*_RC_<0.95 indicates stochastic assembly, with similarity no greater than expected than by chance, *S*_RC_<0.05 significant dissimilarity, *S*_RC_>0.95 significant similarity.

Using the Raup and Crick probability-based similarity index (*S*_RC_), stochasticity in the data was investigated by recording the proportions of microbiota samples taken pairwise, whose compositional similarities where not more or less significant than by chance (*S*_RC_>0.05 and *S*_RC_<0.95). The *S*_RC_ frequency distributions for each microbiome (whole, core and satellite microbiota) were visualized as histograms by binning of *S*_RC_ values (Figure 3). For the whole microbiota, deterministic frequency distributions (significantly similar than expected by chance, *S*_RC_>0.95) decreased through the microbiomes; proventriculus 100%, cloaca 91.8% and faeces 48.9% (Figure 3a). The core microbiota followed similar deterministic patterns of similarity; proventriculus 100%, cloaca 96.0% and faeces 80.0% (Figure 3b). Although the proportion of deterministic associations remained high in the proventriculus satellite microbiota (83%), a large increase in stochastic frequency distributions was observed across the microbiomes; proventriculus 17.1%, cloaca 70.7% and faeces 73.3% (Figure 3c).

**Figure 4:**
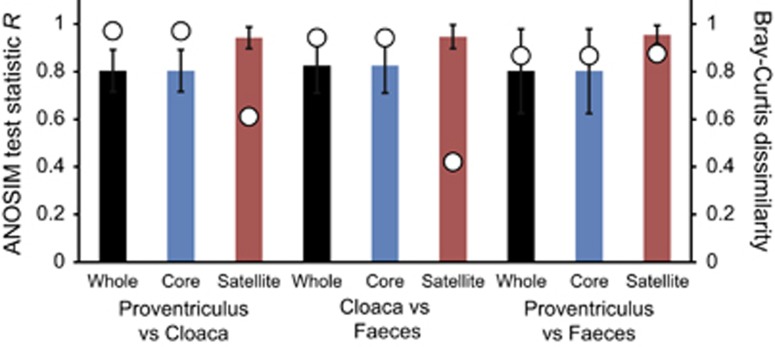
ANOSIM and community dissimilarity for the whole, core and satellite microbiota between microbiomes. Given is the ANOSIM test statistic (*R*, denoted as circles) that two compared groups are significantly different at the *P*<0.05 level (all tests were significant; *P*<0.0001 in each instances). ANOSIM *R-* and *P-*values were generated using the Bray–Curtis measure of similarity. *R* scales from +1 to −1. +1 indicates that all the most similar samples are within the same groups. *R*=0 occurs if the high and low similarities are perfectly mixed and bear no relationship to the group. A value of −1 indicates that the most similar samples are all outside of the groups. Also given are the Bray–Curtis quantitative measures of mean dissimilarity between groups, denoted as columns. Error bars represent the s.d. of the mean dissimilarities.

Analysis of similarities (ANOSIM) tests demonstrated that the whole, core and satellite microbiota compared between microbiomes were highly dissimilar and significantly divergent from each other (*P*<0.0001 in all instances; Figure 4). Similarity of percentages analysis of the whole microbiota was used to identify those taxa that contributed most to the observed dissimilarity between the three microbiomes. Those phylotypes, all core group members, are listed in decreasing order of contribution in Table 2.

Canonical correspondence analysis was used to relate the variability in the distribution of microbiota within microbiomes to intrinsic variables (Table 3). Phenology explained the most variation within the whole and core proventriculus microbiota. Variation in nematode counts significantly explained the most variation in the satellite group, but was not significant for the whole and core. Within the cloacal whole and core microbiota, differences in sex accounted for the most variation, while nematode counts explained the most variation in satellite group; unlike the proventriculus microbiome this was also a significant variable for the whole and core microbiota. Variation within the faecal whole and core microbiota was best explained by variation in nematode counts, with phenology being the better predictor within the satellite group. Further, the percentage variation accounted for by nematode counts was observed to increase through the microbiomes from proventriculus to faeces.

**Figure 5:**
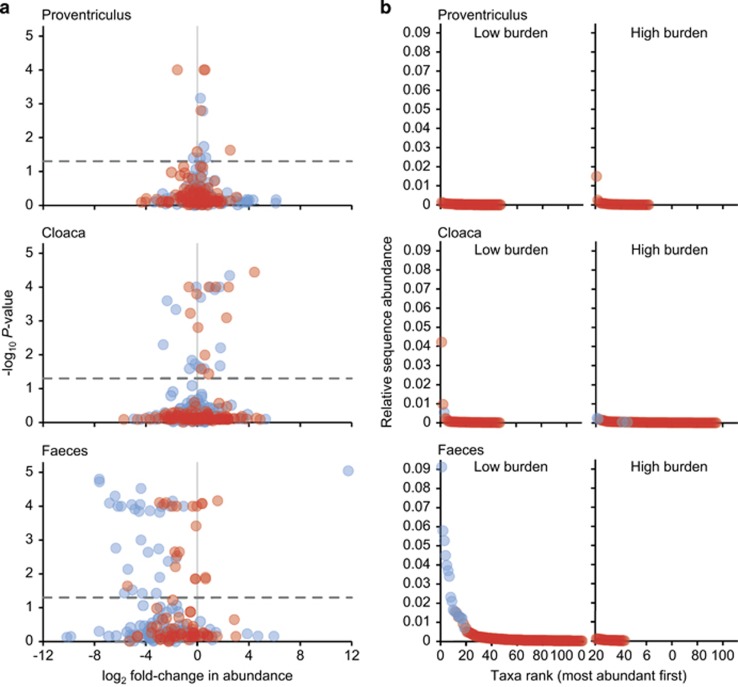
Changes in abundances of individual taxa related to nematode burden in each microbiome. (**a**) Volcano plots displaying fold-changes in relative abundance from low to high nematode burden for core (blue) and satellite (red) taxa. Positive and negative values represent increases and decreases in relative taxa abundance within the high-burden group when compared with the low-burden group. The dashed line depicts *P*=0.05, with taxa above that line having significant fold-changes in abundance, whereas those falling below the line are not significant. Core and satellite taxa that were only detected in low- or high-burden groups are excluded from this analysis as there fold change would be infinity. Instead, (**b**) they are presented in rank-abundance plots for each microbiome. Number of individual hosts by low and high-burden groups for each microbiome are: proventriculus, low *n*=19, high *n*=19; cloaca, low *n*=20, high *n*=19; and faeces, low *n*=6, high *n*=4.

Impact of nematode burden was assessed further at the community and individual taxon level using ANOSIM tests and volcano plots, respectively. For each microbiome, microbiota samples were divided into high and low nematode burdens (high group >median nematode count of 21, and low⩽median count). ANOSIM results revealed no significant differences (*P*>0.05) in whole, core and satellite microbiota within the proventriculus and cloacal microbiomes. Conversely, the faecal whole (*R*=0.504, *P*=0.004), core (*R*=0.504, *P*=0.005) and satellite (*R*=0.268, *P*=0.04) microbiota were significantly related to high and low nematode burden at the *P*<0.05 level. Volcano plots were used to visualise the impact of nematode burden on individual core and satellite taxa within each microbiome by plotting fold change in relative mean abundance against significance (*P*) values from two-sample *t*-tests of differences in relative abundance for each taxon (Figure 5a). Within the proventriculus microbiome, minimal impact was observed with only six core and six satellite taxa demonstrating significant fold-changes in abundance. In the cloacal microbiome the number of taxa that had significant fold-changes in abundance increased, when compared with the proventriculus taxa, with 16 core and 12 satellite taxa. Change in the faecal microbiome taxa was even more pronounced with 30 core and 20 satellite taxa exhibiting predominately negative significant fold-changes in abundance due to differences in nematode burden. Taxa that had zero abundances in either low or high-burden groups were excluded from this analysis as their fold change in abundance would be infinity. However, this would represent a significant difference in abundance. Instead, those taxa are visualised in rank-abundance plots (Figure 5b). Within the proventriculus microbiome 42 taxa were no longer detected in the high-burden group and 47 appeared in the high-burden group, not being previously detected in the low-burden group; these 89 taxa were all members of the proventriculus satellite microbiota. For the cloacal microbiome, 95 taxa (4 core, 91 satellite) were not subsequently detected in the high-burden group, and 47 taxa (1 core, 46 satellite) appeared in the high-burden group. This was more pronounced in the faecal microbiome, with 111 taxa (16 core, 95 satellite) no longer detected in the high-burden group and 23 satellite not previously detected in the low-burden group.

## Discussion

Infection by parasitic helminths of wild vertebrates is ubiquitous in nature, but the impact and interactions of such endoparasites on wild hosts and their gastrointestinal microbiota represents an overlooked but important component of animal population dynamics. Both gut bacteria and helminths affect animal host nutrition and health, and by sharing the same physical niche spaces within the gastrointestinal tract are therefore capable of interacting with each other and the host (Glendinning *et al.*, 2014). Knowledge on such interactions within this three-way relationship is in infancy with initial work predominately focused on human and murine model host, and the effect of endoparasites upon wild host microbiota represents a fundamental knowledge gap in wild animal populations. Here using a novel combination of endoscopy to directly enumerate parasite burden in conjunction with characterisation of gut microbiomes, including the site of infection, we provide one of the first assessments of this three-way relationship in live wild hosts.

The constituent taxa within the gastrointestinal microbiota of the shags sampled were congruent with other vertebrate and avian hosts, with members of the Actinobacteria, Bacteroidetes, Firmicutes, Fusobacteria, Proteobacteria and Tenericutes predominating (Waite and Taylor, 2014) (Table 2 and Supplementary Figure S1). Further, taxa specifically associated with other piscivorous birds were also present, including *Campylobacter canadensis* and *Catellicoccus marimammalium* (Inglis *et al.*, 2007, Lu *et al.*, 2008). Functional partitioning along the avian gastrointestinal tract is believed to be reflected in the composition of associated gut microbiota (Gong *et al.*, 2007; Torok *et al.*, 2008; Godoy-Vitorino *et al.*, 2012; Waite *et al.*, 2012). Assessing functional partitioning without destructive sampling of the host is difficult and most field studies have been limited to focusing on specific regions of the gastrointestinal tract, for example, cloaca or faeces. Here we were able to sample across interconnected microbiomes, with our findings supporting the functional partitioning view (Donaldson *et al.*, 2016), as the proventriculus, cloaca and faecal microbiota were found to be significantly distinct (Figure 4). Further, the observed between microbiota variation was specifically linked to core phylotypes (Table 2).

Deterministic microbiota assembly was found to decrease (Figure 3) with the transition from the upper gastrointestinal tract (proventriculus) to lower tract (cloaca), and finally faeces; with these assembly patterns mainly driven by the abundant non-randomly distributed core microbiota. This is likely a reflection of immigration and establishment within the indigenous microbiota. Within the proventriculus successful immigrant propagation is likely low, as new immigrants would not only have limited immigration routes, mainly through ingestion, but would have to compete with established indigenous populations for resources in a highly selective niche environment, which is anaerobic and highly acidic (Vispo and Karasov, 1997). Higher levels of stochasticity within the faecal samples were indicative of the increased chance of recruitment, as faeces are formed and pass through the gastrointestinal tract, reflected by increasing high levels of stochasticity in the satellite microbiota. Cloacal samples represented an intermediary of the two having a more favourable environment than the proventriculus and potentially increased immigration via defecation and copulation. It has previously been shown in a kittiwake population that copulation affected diversity and composition of female cloacal microbiomes, serving as a bacterial exchange mechanism with mated pairs, which had similar microbiota and became divergent when insemination was blocked (White *et al.*, 2010).

Congruent with previous work (Burthe *et al.*, 2013), host sex and nematode burden were significantly correlated, with males having higher nematode counts than females. Further, host sex was a significant explanatory variable when direct ordination was used to explain community variation (Table 3). A common finding in ecological studies is that males are more heavily prone to parasitism than females in both mammalian (Schalk and Forbes, 1997; Moore and Wilson, 2002) and avian hosts (Poulin, 1996; McCurdy *et al.*, 1998; Stewart and Merrill, 2015). For instance, physiological differences such as androgen concentrations, have been demonstrated as being immunosuppressive in many bird species (Duffy *et al.*, 2000; Evans *et al.*, 2000; Deviche and Cortez, 2005; Sandell *et al.*, 2009) and the high prevalence of immune response associated genes linked to sexual bias in parasitism (Bianchi *et al.*, 2012; Markle and Fish, 2014). In addition, ecological strategies also hold a role through both sexual forces and sexual bias behaviour. The immunocompetence handicap hypothesis suggests that many male characteristics are controlled by testosterone, at the cost of immunosuppression (Folstad and Karter, 1992). Yet the direct effect of testosterone to gastrointestinal tract microbiota is less understood. In the human gut it appears that the microbiota can influence sex hormone levels, which can help modulate the immune system (Markle *et al.*, 2013). Further, *Clostridium scindens* within the human gut microbiota has been observed to likely possess the ability to convert glucocorticoids into androgens (Ridlon *et al.*, 2013). Clearly the effect of androgens upon gut microbiota and *vice versa* is an area which warrants further investigation.

Within the current study, significant sex related differences in bacterial diversity were detected in the cloacal and faecal microbiomes; with diversity higher in male cloacal samples and, conversely higher in female faecal samples (Figure 2). One reason for lower diversity in the female cloacal microbiota may be a response to egg laying. Microbial infection during early life stages is a key factor of mortality in oviparous vertebrates and therefore, the minimisation of exposure to pathogens would be highly beneficial. Bird species have been reported to achieve this through incubation and adhesion of antimicrobial producing bacteria to eggs (Shawkey *et al.*, 2009; Martin-Vivaldi *et al.*, 2014). Although based on lower sample numbers, indicative of the difficulties of obtaining faecal samples in the field (Burthe *et al.*, 2013), lower bacterial diversity in male faecal samples may be due to the influence of higher parasite infection in males. However, the difference between male and female cloacal and faecal microbiomes would not have been revealed without sampling distinct microbiomes along the gastrointestinal tract (Donaldson *et al.*, 2016). Interestingly, host age and phenology also appear as important factors in explaining microbiota variation (Table 3), and would warrant further study, as from previous work we found that burden increases over the breeding season, with late breeders more susceptible to helminth infection or its associated costs, potentially because they tend to be younger, less experienced, and less capable of mounting effective immune responses (Daunt *et al.*, 1999; Reed *et al.*, 2008; Burthe *et al.*, 2013).

It has been posited that helminths have the capacity to maintain higher gut microbiota diversity and may represent gut homoeostasis (Kreisinger *et al.*, 2015). Conversely, no significant within microbiome relationships between burden and diversity were observed in this study. However, whole and core microbiota diversity between microbiomes was observed to significantly increase away from the site of infection (Figure 2). In addition, the burden of *C*. *ruldophii* infection was significantly associated with changes in microbiota composition. Direct ordination indicated that the effect became more prominent away from the site of infection and increasingly accounted for variation in the whole, core and satellite microbiota within the cloacal and faecal microbiomes, respectively (Table 3); and may also have been a contributing factor to the increased stochastic microbiota assembly through the gastrointestinal tract (Figure 3). The increasing dysbiosis in composition occurring away from the site of infection, in part, may be explained by the nematodes inducing an anti-inflammatory environment in the proventriculus, diverting immune responses away from themselves (Maizels *et al.*, 2004; Kreisinger *et al.*, 2015) and also the contribution of additional competing bacterial populations to the host microbiota from their own faecal microbiota, more prominently when parasite burdens were high. This was further emphasised when within microbiome samples were partitioned into high and low parasite burden groups, with an increasing dysbiosis in microbiota due to high-burden predominately resulting in negative shifts in taxa abundance in core and satellite taxa (Figure 5). Strikingly, faecal samples from birds with high parasite burdens contained significantly higher abundances (>10-fold change) of *Cetobacterium somerae* like phylotypes. *Cetobacterium somerae* is known to be highly prevalent within fish gut microbiomes (Larsen *et al.*, 2014), and as fish prey are the intermediate host in the *C. rudolphii* life cycle, the close association with high parasite burden may be indicative of shared transmission between *C. rudolphii* and *C. somerae*. A recommendation for future studies would be to additionally characterise the nematode microbiota to further elucidate microbial interchange from the parasite to the host.

In conclusion, we found that nematode burden appears to have a progressively strong downstream affect in significantly distinct microbiomes through the gastrointestinal tract, observing an increasingly pronounced dysbiosis in microbiota when partitioned into low and high parasite burden groups. This gives a clear indication of interactions between intestinal parasites and gastrointestinal microbiota within a wild host, which would have not been elucidated without a combination of sampling multiple microbiomes through the gastrointestinal tract and direct enumeration of parasites at the site of infection. In the study host, infection is primarily by a single helminth species and localised to the lower oesophagus and proventriculus only. In other hosts, co-infection by multiple helminths can occur (for example, Kreisinger *et al.*, 2015). As such, and where applicable, future studies should consider the co-infection interactions with the gut microbiota. Taken together, this cross-sectional study provides a foundation to better understand the mechanisms that underpin the three-way relationship between the wild host, helminth and microbiota. To address the mechanistic basis of these interactions, future studies will be able to uniquely investigate the longitudinal short-term (current breeding performance), and long-term (future survival and breeding) implications for the population dynamics, coupled with host immune responses and alterations to metabolites produced by the microorganisms and helminths through the gastrointestinal tract microbiomes.

## Figures and Tables

**Table 1:** Summary of intrinsic variables in the adult shags sampled

*Variable*			
Individuals	Total=40	Females=18	Male=22
Mass (g)	Min=1460	Max=2130	Mean=1791.7±178.5 (s.d.)
Age (years)	Min=3	Max=17	Mean=7.8±4.3 (s.d.)
Number of chicks fledged^a^	Min=1	Max=3	Mean=2.25±0.67
Phenology^b^	23 early	17 late	
Nematode counts	Min=3	Max=45	Mean=24.8±11.7 (s.d.)

a Total number of chicks fledged per bird—a measure of breeding success.

b Brood hatched at a date, before (early) or after (late) the median hatch date for the colony.

**Table 2:** Similarity of percentages (SIMPER) analysis of community dissimilarity (Bray–Curtis) between whole microbiota of proventriculus, faecal and cloacal samples

*Phylum*	*Taxon*	*Av. Dissim.*	*Contrib. %*	*Cum. %*	*% Mean abundance*		
					*Proventriculus*	*Cloaca*	*Faeces*
Proteobacteria	*Suttonella* 61%	8.34	10.34	10.34	20.3	0.32	2.86
Fusobacteria	*Fusobacterium* 96%	7.22	8.95	19.29	17.5	1.41	9
Actinobacteria	*Corynebacterium falsenii* 93%	5.35	6.63	25.92	1.07	13.3	0.62
Firmicutes	*Mageeibacillus indolicus* 65%	4.03	5	30.92	1.4	10.8	1.28
Firmicutes	*Clostridium* 99%	3.84	4.76	35.69	0.28	4.12	15.9
Fusobacteria	*Streptobacillus moniliformis* 97%*	3.39	4.21	39.89	9.17	1.43	1.8
Firmicutes	*Clostridium sordellii* 100%	2.76	3.43	43.32	0.004	0.06	16.1
Proteobacteria	*Alcaligenaceae* 97%*	2.76	3.43	46.74	0.83	7.01	0.97
Proteobacteria	*Campylobacter canadensis* 100%	2.36	2.93	49.67	0.07	5.42	2.07
Actinobacteria	*Varibaculum* 67%	2.15	2.67	52.34	0.11	5.17	0.91
Actinobacteria	*Microbispora rosea* 97%*	2.1	2.6	54.94	6.04	1.98	0.68
Firmicutes	*Lactobacillus aviarius* 100%	1.98	2.46	57.4	0.004	0.39	11
Proteobacteria	*Sutterella morbirensis* 100%	1.83	2.27	59.67	4.51	0.11	0.33
Actinobacteria	*Varibaculum* 67%	1.8	2.23	61.91	1.29	5.1	0.48
Firmicutes	*Catellicoccus marimammalium* 100%	1.77	2.2	64.1	0.02	0.68	8.79
Firmicutes	*Coprococcus* 79%	1.68	2.08	66.18	2.77	5.55	1.85
Firmicutes	*Finegoldia* 94%	1.54	1.91	68.09	0.15	3.75	0.19
Bacteroidetes	*Rikenellaceae* RC9_gut group 100%	1.47	1.82	69.9	3.74	0.66	0.26
Firmicutes	*Peptoniphilus* 100%	1.43	1.77	71.68	0.15	3.49	0.22
Bacteroidetes	*Porphyromonas canoris* 100%	1.24	1.54	73.21	3.33	2.24	0.74
Tenericutes	*Acholeplasma* 97%*	1.22	1.52	74.73	3.05	0.19	0.47
Firmicutes	*Veillonella dispar* 97%*	1.16	1.44	76.17	2.55	3.6	0.64
Firmicutes	*Veillonella* 97%	1.16	1.44	77.61	3.07	0.43	0.57
Firmicutes	*Clo. colinum* 100%	0.99	1.23	78.84	0.43	0.73	4.92
Firmicutes	*Megamonas* 100%	0.93	1.15	79.99	2.23	0.1	0.45
Actinobacteria	*Actinomyces europaeus* 100%	0.89	1.1	81.09	0.12	2.14	0.12
Bacteroidetes	*Ornithobacterium rhinotracheale* 100%	0.86	1.07	82.16	2.1	0.04	0.27
Bacteroidetes	*Parabacteroides* 97%*	0.84	1.04	83.2	0.04	2.03	0.16
Firmicutes	*Tissierellaceae* 97%*	0.79	0.98	84.18	0.08	1.92	0.26
Firmicutes	*Peptococcus niger* 100%	0.73	0.9	85.08	1.67	1.14	0.2
Firmicutes	*Peptostreptococcus* 95%	0.72	0.89	85.97	1.83	0.38	0.76
Actinobacteria	*Brevibacterium* 99%	0.67	0.83	86.8	0.06	1.61	0.06
Actinobacteria	*Propionibacteriaceae* 97%	0.66	0.81	87.62	1.75	0.69	0.23
Bacteroidetes	*Petrimonas* 78%	0.61	0.75	88.37	1.47	0.05	0.07
Actinobacteria	*Arthrobacter* 66%	0.57	0.71	89.08	0.3	1.39	0.21
Fusobacteria	*Cetobacterium somerae* 65%	0.57	0.71	89.78	0.004	0.03	3.25
Tenericutes	*Mycoplasma gallopavonis* 60%	0.56	0.7	90.48	1.42	0.11	0.22
Actinobacteria	*Pseudoclavibacter bifida* 97%*	0.55	0.68	91.16	0.05	1.33	0.07
Proteobacteria	*Psychrobacter* 92%	0.43	0.53	91.7	0.03	1.04	0.11

Abbreviation: SIMPER, similarity of percentages.

Given is the mean % abundance of sequences for bacterial taxa only across the samples each was observed to occupy and average dissimilarity between sample (overall mean=80.7%). Percentage contribution is the mean contribution divided by the mean dissimilarity across samples. The list of taxa is not exhaustive, so cumulative % does not sum to 100%. The taxonomic identity with the highest resolution (for key taxa) determined through phylogenetic association and/or taxonomic assignment* are reported here. Percentage values represent bootstrap or taxonomic assignment* support (%).

**Table 3:** CCA for determination of percent variation in the whole, common, and satellite microbiota within each of the three microbiomes by demographic variables significant at the *P*<0.05 level

*% of variation*	*Proventriculus*			*Cloaca*			*Faeces*		
	*Whole*	*Core*	*Satellite*	*Whole*	*Core*	*Satellite*	*Whole*	*Core*	*Satellite*
Age (years)	6.3	6.3	3.2	2.8	2.8	3.8	11.0	11.0	13.6
Host sex	5.4	5.4	4.2	12.0	12.0	2.3	11.4	11.4	15.5
Nematode burden	—	—	7.1	4.9	4.9	9.5	21.0	21.1	9.3
Phenology	13.6	13.6	3.7	—	—	—	8.1	8.1	17.3
Undetermined	74.7	74.7	81.9	80.2	80.2	84.5	48.5	48.5	44.4

Abbreviation: CCA, canonical correspondence analyses.
