# Supplementary material for: Helminth burden and ecological factors associated with alterations in wild host gastrointestinal microbiota
Source: ISME J. 2016 Dec 16;11(3):663–75. doi: 10.1038/ismej.2016.153 (PMC5322305; doi:10.1038/ismej.2016.153)
Supplement: Supplementary Information [file ismej2016153x1.docx]

**Helminth burden associated with alterations in wild host gastrointestinal microbiota**

Lindsay K. Newbold^1†^, Sarah J. Burthe^2†^, Anna E. Oliver^1^, Hyun S. Gweon^1^, Christopher J. Barnes^1,3^, Francis H.J. Daunt^2^, and Christopher J. van der Gast^1^*

*^1^ NERC Centre for Ecology & Hydrology, Wallingford, OX10 8BB, UK*

*^2^ NERC Centre for Ecology & Hydrology, Edinburgh, EH26 0QB, UK*

*^3^ National History Museum of Denmark, 83 Sølvgade, University of Copenhagen, Copenhagen, 1800, Denmark*

** Correspondence: E-mail cjvdg@ceh.ac.uk*

**Supplementary Material**

*File content*

This file contains additional information about endoscopy procedures, molecular methods and sequence analysis.

*Endoscopy*

Endoscopy of conscious adult shags was undertaken, as previously described ([Burthe et al 2013](#_ENREF_2)), using a 103 cm long, 9 mm diameters Olympus UK Ltd, Southend-on-Sea, UK GIF-PQ20 gastroscope with a 2.8 mm channel for air and water, a 100^o^ field of view and a 150 W halogen light source (VES) with inbuilt air and water pump, powered by a portable EU10i generator (Honda, Swindon, UK). The gastroscope was flexible through 210/90^o^ vertically and 100/100^o^ horizontally. A compact camera (Xion) connected to the endoscope was used to view and record video images on a laptop using XION DiVASMini image software (Xion Medical, Berlin, Germany).

To ensure that birds had empty stomachs, endoscopy was undertaken between 03:30 and 07:30 hours, before shags left for their first foraging trip of the day. An assistant placed a cloth over the bird’s eyes to reduce stress and held the neck stretched out on a cushion to prevent movement, with the beak open approximately 2cm. The endoscope was lubricated with KY jelly (Johnson & Johnson, New Brunswick, New Jersey, USA) and gently inserted down the oesophagus into the stomach up to a length of 50 cm (the base of the proventriculus) from the tip of the beak (measured using graduations on the endoscope). Slight inflation with air helped introduce the endoscope into the stomach and facilitated effective examination. Once a clear view was obtained on the laptop, video recording was started and the scope was pulled out slowly and steadily, enabling nematodes to be counted. The endoscope was cleaned and disinfected between birds using a high-level disinfectant TriGene (Medichem International Ltd, Sevenoaks, UK) wipe. The channels were cleaned with a soft brush and flushed with diluted sterilising fluid (Milton, Proctor & Gamble, Weybridge, UK) followed by deionised water. At the end of each session (average 6.5 birds per session) the endoscope was soaked and cleaned with MedEzyme cleaner and MedDis disinfectant (Medichem International Ltd, Sevenoaks, UK) followed by deionised water. Ideally in clinical setting, the endoscope would be soaked for 20-45 min in enzymatic cleaner and disinfectant between patients. However, this approach was not practical in the field and hence we adopted a procedure based on veterinary advice that reduced the risk of disease transmission to an acceptable level given the non-sterile, challenging field conditions. To ensure accurate sampling of the proventriculus lining for microbiota analyses, samples were gained by taking swabs from the recessed camera lens of the gastroscope.

*DNA extraction and sequencing*

Shag gut microbiome DNA was extracted from sample swabs using the PowerSoil®-htp 96 Well Soil DNA Isolation Kit, (Mobio Laboratories Inc., Carlsbad, CA). Briefly, swab tips were excised into PowerSoil®-htp bead plates containing PowerSoil®-htp bead solution and solution C1. These plates were incubated at 60ºC for 20 mins, then horizontally vortexed for a further 20 mins at 2000rpm. Following these additional lysis steps manufacturers recommended protocol was followed. Approximately 20-30 ng of template DNA was amplified using Q5**®** high-fidelity DNA polymerase (New England Biolabs, Hitchin, UK) each with a unique dual-index barcode primer combination ([Kozich et al 2013](#_ENREF_7)). Individual PCR reactions employed 25 cycles of an initial 30 sec, 98ºC denaturation step, followed by annealing phase for 30 sec at 53ºC, and final extension step lasting 90 secs at 72 ºC. Primers were based upon the universal primer sequence 341F (5’- CCATCTCATCCCTGCGTGTCTCCGACTCAG) and 806R (5’- GCTGCCTCCCGTAGGAGT). An amplicon library consisting of ~550 bp amplicons spanning the V3-V4 hypervariable regions of the 16S rRNA gene, was generated from gel purified pooled products of 4 replicate PCR reactions, per sample. Quantification was performed on an Agilent 2200 TapeStation system and concentration calculated using a SYBR green quantitative PCR (qPCR) assay with primers specific to the Illumina adapters (Kappa, Anachem) an eqimolar mix of PCR products was prepared and diluted to 20pM in dH_2_0.

The 16S library was sequenced at a concentration of 5.4 pM with a 0.6 pM addition of an Illumina generated PhiX control library. Sequencing runs, generating 2 x 300 bp, reads were performed on an Illumina MiSeq using V3 chemistry. The read 1 (R1), read 2 (R2) and index sequencing primers used were also 16S specific: R1 = sequence of the combined pad, linker and 341F; R2 = sequence of the combined pad, linker and 806R; I = reverse compliment of the R2 primer (See Table S1).

*Sequence analysis*

Sequenced paired-end reads were joined using PEAR (Zhang et al. 2014), quality filtered using FASTX tools (Hannon, http://hannonlab.cshl.edu) and chimeras were identified and removed with ChimeraSlayer ([Haas et al 2011](#_ENREF_6)). The sequences were clustered into operational taxonomic units (OTUs) with UCLUST ([Edgar 2010](#_ENREF_5)) as part of the QIIME package ([Caporaso et al 2010](#_ENREF_3)) and representative sequences were selected (pick_rep_set.py, QIIME). The taxonomy of representatives was determined by QIIME's UCLUST consensus taxonomy assigner (assign_taxonomy.py, QIIME) using the Greengenes database release 13_2 ([McDonald et al 2012](#_ENREF_8)). Resultant OTUs were combined to create phylotypes, associated at the 97% identity similarity cut-off, which roughly corresponds to a species/genus level ([Stackebrandt and Goebel 1994](#_ENREF_12)). As an additional measure the identity of reference sequences from the most abundant OTU within each phylotype was corroborated using phylogenetic association**.** To control for putative kit contaminants process negative control samples, were included in analyses and potential false positives removed ([Salter et al 2014](#_ENREF_11)).

Experimental sequences, sequences from a selection of published sequences and putative nearest neighbours (selected by SILVA) were aligned using **SINA** (SILVA INcremental Aligner) web aligner ([Pruesse et al 2007](#_ENREF_10)). Accession number of published sequences is given in Table S2. The final alignment contained 498 sequences and 590 nucleotide positions after common gaps were removed. Optimal likelihood settings were determined to be TIM2+-I+-G through the implementation of the Akaike Information Criterion ([Akaike 1998](#_ENREF_1)) in JModelTest V2 ([Darriba et al 2012](#_ENREF_4), [Posada 2008](#_ENREF_9)). PAUP4b8 (Swofford, 2002) was used to generate a Neighbour Joining (NJ) tree using the likelihood criterion (with optimal settings), and bootstrap support values for 1000 replicates. The resultant tree was used to determine the taxonomic affiliation of each phylotype and identity corroborated given via inclusion in the nearest supported cluster (at 70 % bootstrap value or higher). The raw sequence data reported in this study have been deposited in the European Nucleotide Archive under study accession number PRJEB10889. The relevant barcode information for each sample is shown in Table S1.

**References**

Akaike H (1998). Information Theory and an Extension of the Maximum Likelihood Principle. In: Parzen E, Tanabe K, Kitagawa G (eds). *Selected Papers of Hirotugu Akaike*. Springer New York. pp 199-213.

Burthe S, Newell MA, Goodman G, Butler A, Bregnballe T, Harris E *et al* (2013). Endoscopy as a novel method for assessing endoparasite burdens in free-ranging European shags (*Phalacrocorax aristotelis*). *Methods Ecol Evol* **4:** 207-216.

Caporaso JG, Kuczynski J, Stombaugh J, Bittinger K, Bushman FD, Costello EK *et al* (2010). QIIME allows analysis of high-throughput community sequencing data. *Nature methods* **7:** 335-336.

Darriba D, Taboada GL, Doallo R, Posada D (2012). jModelTest 2: more models, new heuristics and parallel computing. *Nat Meth* **9:** 772-772.

Edgar RC (2010). Search and clustering orders of magnitude faster than BLAST. *Bioinformatics* **26:** 2460-2461.

Haas BJ, Gevers D, Earl AM, Feldgarden M, Ward DV, Giannoukos G *et al* (2011). Chimeric 16S rRNA sequence formation and detection in Sanger and 454-pyrosequenced PCR amplicons. *Genome Research* **21:** 494-504.

Kozich JJ, Westcott SL, Baxter NT, Highlander SK, Schloss PD (2013). Development of a dual-index sequencing strategy and curation pipeline for analyzing amplicon sequence data on the MiSeq Illumina sequencing platform. *Appl Environ Microbiol* **79:** 5112-5120.

McDonald D, Price MN, Goodrich J, Nawrocki EP, DeSantis TZ, Probst A *et al* (2012). An improved Greengenes taxonomy with explicit ranks for ecological and evolutionary analyses of bacteria and archaea. *ISME J* **6:** 610-618.

Posada D (2008). jModelTest: Phylogenetic Model Averaging. *Molecular Biology and Evolution* **25:** 1253-1256.

Pruesse E, Quast C, Knittel K, Fuchs BM, Ludwig W, Peplies J *et al* (2007). SILVA: a comprehensive online resource for quality checked and aligned ribosomal RNA sequence data compatible with ARB. *Nucleic Acids Research* **35:** 7188-7196.

Salter SJ, Cox MJ, Turek EM, Calus ST, Cookson WO, Moffatt MF *et al* (2014). Reagent and laboratory contamination can critically impact sequence-based microbiome analyses. *BMC Biol* **12:** 87

Stackebrandt E, Goebel BM (1994). Taxonomic Note: A Place for DNA-DNA Reassociation and 16S rRNA Sequence Analysis in the Present Species Definition in Bacteriology. *International Journal of Systematic Bacteriology* **44:** 846-849.
