# Supplementary material for: Helminth burden and ecological factors associated with alterations in wild host gastrointestinal microbiota
Source: ISME J. 2016 Dec 16;11(3):663–75. doi: 10.1038/ismej.2016.153 (PMC5322305; doi:10.1038/ismej.2016.153)
Supplement: Supplementary Figure S1 [file ismej2016153x2.pdf]

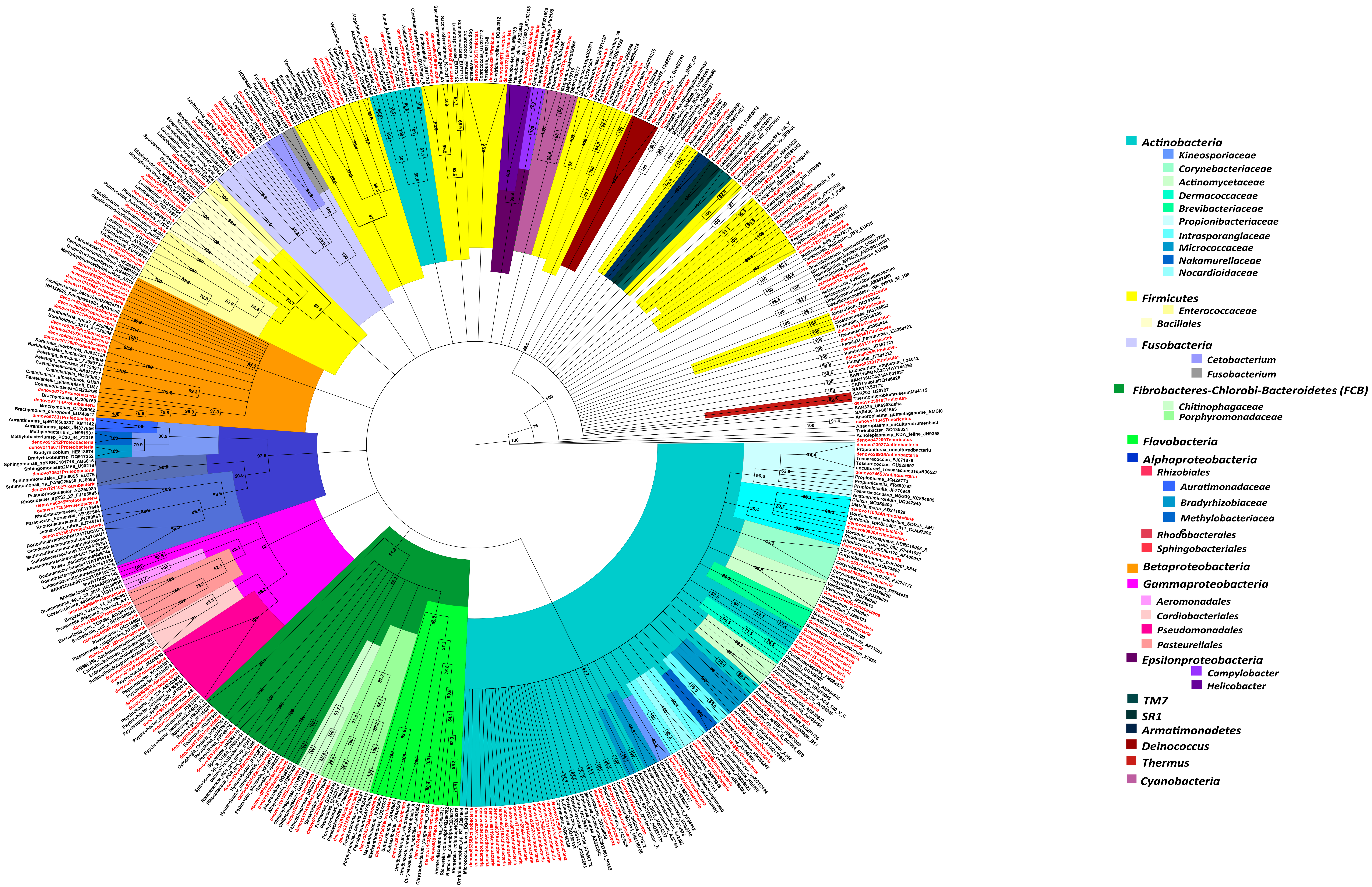

Figure S1: A 50% Majority Rule Consensus Tree of dominant prokaryotic phylotypes identified within the European shag gut microbiome. Sequence identity is inferred by neighbour joining distance criterion under TIM2+I+G model. Bootstap support form 1000 replicates shown at nodes. Phyla/subphyla are highlighted as follows: Dark Blue = Alphaproteobacteria, Pink= Gammaproteobacteria, Orange = Betaproteobacteria, Purple = Epsilonproteobacteria, Green = Fibrobacteres-Chlorobi-Bacteroidetes (FCB), Yellow = Firmicutes, Blue/Grey = Fusobacteria, Light Blue = Actinobacteria. For more specific taxonomic grouping refer to key. Sequences identified in this study are written in red.
