# Supplementary material for: Helminth burden and ecological factors associated with alterations in wild host gastrointestinal microbiota
Source: ISME J. 2016 Dec 16;11(3):663–75. doi: 10.1038/ismej.2016.153 (PMC5322305; doi:10.1038/ismej.2016.153)
Supplement: Supplementary Table S1 [file ismej2016153x3.docx]

**Table S1** Bird identification number, sample type and dual-indexed barcode primer combination.

| Bird ID | Sample type | Primer Name | Primer sequence |
| --- | --- | --- | --- |
| 21955 | Cloacal | V3.F.SA501  V4.R.SB710 | AATGATACGGCGACCACCGAGATCTACACATCGTACGTATGGTAATTGGCCTACGGGAGGCAGCAG  CAAGCAGAAGACGGCATACGAGATTACGAGCAAGTCAGTCAGCCGGACTACHVGGGTWTCTAAT |
|  | Endoscope | V3.F.SA508  V4.R.SB709 | AATGATACGGCGACCACCGAGATCTACACGACACCGTTATGGTAATTGGCCTACGGGAGGCAGCAG  CAAGCAGAAGACGGCATACGAGATGTATACGCAGTCAGTCAGCCGGACTACHVGGGTWTCTAAT |
| 22979 | Endoscope | V3.F.SA501  V4.R.SB712 | AATGATACGGCGACCACCGAGATCTACACATCGTACGTATGGTAATTGGCCTACGGGAGGCAGCAG  CAAGCAGAAGACGGCATACGAGATTCGCTACGAGTCAGTCAGCCGGACTACHVGGGTWTCTAAT |
|  | Cloacal | V3.F.SA508  V4.R.SB711 | AATGATACGGCGACCACCGAGATCTACACGACACCGTTATGGTAATTGGCCTACGGGAGGCAGCAG  CAAGCAGAAGACGGCATACGAGATTCAGCGTTAGTCAGTCAGCCGGACTACHVGGGTWTCTAAT |
| 23165 | Cloacal | V3.F.SA506  V4.R.SB709 | AATGATACGGCGACCACCGAGATCTACACCGTGAGTGTATGGTAATTGGCCTACGGGAGGCAGCAG  CAAGCAGAAGACGGCATACGAGATGTATACGCAGTCAGTCAGCCGGACTACHVGGGTWTCTAAT |
|  | Endoscope | V3.F.SA507  V4.R.SB709 | AATGATACGGCGACCACCGAGATCTACACGGATATCTTATGGTAATTGGCCTACGGGAGGCAGCAG  CAAGCAGAAGACGGCATACGAGATGTATACGCAGTCAGTCAGCCGGACTACHVGGGTWTCTAAT |
| 23244 | Cloacal | V3.F.SA503  V4.R.SB704 | AATGATACGGCGACCACCGAGATCTACACTAGCGAGTTATGGTAATTGGCCTACGGGAGGCAGCAG  CAAGCAGAAGACGGCATACGAGATCATAGAGAAGTCAGTCAGCCGGACTACHVGGGTWTCTAAT |
|  | Endoscope | V3.F.SA504  V4.R.SB704 | AATGATACGGCGACCACCGAGATCTACACCTGCGTGTTATGGTAATTGGCCTACGGGAGGCAGCAG  CAAGCAGAAGACGGCATACGAGATCATAGAGAAGTCAGTCAGCCGGACTACHVGGGTWTCTAAT |
| 23449 | Endoscope | V3.F.SA501  V4.R.SB702 | AATGATACGGCGACCACCGAGATCTACACATCGTACGTATGGTAATTGGCCTACGGGAGGCAGCAG  CAAGCAGAAGACGGCATACGAGATATACTTCGAGTCAGTCAGCCGGACTACHVGGGTWTCTAAT |
| 23702 | Faecal | V3.F.SA501  V4.R.SB703 | AATGATACGGCGACCACCGAGATCTACACATCGTACGTATGGTAATTGGCCTACGGGAGGCAGCAG  CAAGCAGAAGACGGCATACGAGATAGCTGCTAAGTCAGTCAGCCGGACTACHVGGGTWTCTAAT |
|  | Endoscope | V3.F.SA502  V4.R.SB703 | AATGATACGGCGACCACCGAGATCTACACACTATCTGTATGGTAATTGGCCTACGGGAGGCAGCAG  CAAGCAGAAGACGGCATACGAGATAGCTGCTAAGTCAGTCAGCCGGACTACHVGGGTWTCTAAT |
|  | Cloacal | V3.F.SA508  V4.R.SB702 | AATGATACGGCGACCACCGAGATCTACACGACACCGTTATGGTAATTGGCCTACGGGAGGCAGCAG  CAAGCAGAAGACGGCATACGAGATATACTTCGAGTCAGTCAGCCGGACTACHVGGGTWTCTAAT |
| 24562 | Endoscope | V3.F.SA506  V4.R.SB712 | AATGATACGGCGACCACCGAGATCTACACCGTGAGTGTATGGTAATTGGCCTACGGGAGGCAGCAG  CAAGCAGAAGACGGCATACGAGATTCGCTACGAGTCAGTCAGCCGGACTACHVGGGTWTCTAAT |
|  | Cloacal | V3.F.SA507  V4.R.SB712 | AATGATACGGCGACCACCGAGATCTACACGGATATCTTATGGTAATTGGCCTACGGGAGGCAGCAG  CAAGCAGAAGACGGCATACGAGATTCGCTACGAGTCAGTCAGCCGGACTACHVGGGTWTCTAAT |
| 24869 | Cloacal | V3.F.SA501  V4.R.SB708 | AATGATACGGCGACCACCGAGATCTACACATCGTACGTATGGTAATTGGCCTACGGGAGGCAGCAG  CAAGCAGAAGACGGCATACGAGATGGTACTATAGTCAGTCAGCCGGACTACHVGGGTWTCTAAT |
|  | Faecal | V3.F.SA507  V4.R.SB707 | AATGATACGGCGACCACCGAGATCTACACGGATATCTTATGGTAATTGGCCTACGGGAGGCAGCAG  CAAGCAGAAGACGGCATACGAGATGCGCACGTAGTCAGTCAGCCGGACTACHVGGGTWTCTAAT |
|  | Endoscope | V3.F.SA508  V4.R.SB707 | AATGATACGGCGACCACCGAGATCTACACGACACCGTTATGGTAATTGGCCTACGGGAGGCAGCAG  CAAGCAGAAGACGGCATACGAGATGCGCACGTAGTCAGTCAGCCGGACTACHVGGGTWTCTAAT |
| 25108 | Cloacal | V3.F.SA504  V4.R.SB702 | AATGATACGGCGACCACCGAGATCTACACCTGCGTGTTATGGTAATTGGCCTACGGGAGGCAGCAG  CAAGCAGAAGACGGCATACGAGATATACTTCGAGTCAGTCAGCCGGACTACHVGGGTWTCTAAT |
|  | Endoscope | V3.F.SA505  V4.R.SB702 | AATGATACGGCGACCACCGAGATCTACACTCATCGAGTATGGTAATTGGCCTACGGGAGGCAGCAG  CAAGCAGAAGACGGCATACGAGATATACTTCGAGTCAGTCAGCCGGACTACHVGGGTWTCTAAT |
| 25482 | Cloacal | V3.F.SA505  V4.R.SB711 | AATGATACGGCGACCACCGAGATCTACACTCATCGAGTATGGTAATTGGCCTACGGGAGGCAGCAG  CAAGCAGAAGACGGCATACGAGATTCAGCGTTAGTCAGTCAGCCGGACTACHVGGGTWTCTAAT |
|  | Endoscope | V3.F.SA506  V4.R.SB711 | AATGATACGGCGACCACCGAGATCTACACCGTGAGTGTATGGTAATTGGCCTACGGGAGGCAGCAG  CAAGCAGAAGACGGCATACGAGATTCAGCGTTAGTCAGTCAGCCGGACTACHVGGGTWTCTAAT |
| 25896 | Cloacal | V3.F.SA505  V4.R.SB704 | AATGATACGGCGACCACCGAGATCTACACTCATCGAGTATGGTAATTGGCCTACGGGAGGCAGCAG  CAAGCAGAAGACGGCATACGAGATCATAGAGAAGTCAGTCAGCCGGACTACHVGGGTWTCTAAT |
|  | Endoscope | V3.F.SA506  V4.R.SB704 | AATGATACGGCGACCACCGAGATCTACACCGTGAGTGTATGGTAATTGGCCTACGGGAGGCAGCAG  CAAGCAGAAGACGGCATACGAGATCATAGAGAAGTCAGTCAGCCGGACTACHVGGGTWTCTAAT |
| 27423 | Faecal | V3.F.SA504  V4.R.SB707 | AATGATACGGCGACCACCGAGATCTACACCTGCGTGTTATGGTAATTGGCCTACGGGAGGCAGCAG  CAAGCAGAAGACGGCATACGAGATGCGCACGTAGTCAGTCAGCCGGACTACHVGGGTWTCTAAT |
|  | Endoscope | V3.F.SA505  V4.R.SB707 | AATGATACGGCGACCACCGAGATCTACACTCATCGAGTATGGTAATTGGCCTACGGGAGGCAGCAG  CAAGCAGAAGACGGCATACGAGATGCGCACGTAGTCAGTCAGCCGGACTACHVGGGTWTCTAAT |
| 27424 | Cloacal | V3.F.SA506  V4.R.SB707 | AATGATACGGCGACCACCGAGATCTACACCGTGAGTGTATGGTAATTGGCCTACGGGAGGCAGCAG  CAAGCAGAAGACGGCATACGAGATGCGCACGTAGTCAGTCAGCCGGACTACHVGGGTWTCTAAT |
| 27763 | Endoscope | V3.F.SA507  V4.R.SB706 | AATGATACGGCGACCACCGAGATCTACACGGATATCTTATGGTAATTGGCCTACGGGAGGCAGCAG  CAAGCAGAAGACGGCATACGAGATCTCGTTACAGTCAGTCAGCCGGACTACHVGGGTWTCTAAT |
|  | Cloacal | V3.F.SA508  V4.R.SB706 | AATGATACGGCGACCACCGAGATCTACACGACACCGTTATGGTAATTGGCCTACGGGAGGCAGCAG  CAAGCAGAAGACGGCATACGAGATCTCGTTACAGTCAGTCAGCCGGACTACHVGGGTWTCTAAT |
| 28625 | Faecal | V3.F.SA501  V4.R.SB707 | AATGATACGGCGACCACCGAGATCTACACATCGTACGTATGGTAATTGGCCTACGGGAGGCAGCAG  CAAGCAGAAGACGGCATACGAGATGCGCACGTAGTCAGTCAGCCGGACTACHVGGGTWTCTAAT |
|  | Cloacal | V3.F.SA502  V4.R.SB707 | AATGATACGGCGACCACCGAGATCTACACACTATCTGTATGGTAATTGGCCTACGGGAGGCAGCAG  CAAGCAGAAGACGGCATACGAGATGCGCACGTAGTCAGTCAGCCGGACTACHVGGGTWTCTAAT |
|  | Endoscope | V3.F.SA503  V4.R.SB707 | AATGATACGGCGACCACCGAGATCTACACTAGCGAGTTATGGTAATTGGCCTACGGGAGGCAGCAG  CAAGCAGAAGACGGCATACGAGATGCGCACGTAGTCAGTCAGCCGGACTACHVGGGTWTCTAAT |
| 29934 | Cloacal | V3.F.SA504  V4.R.SB703 | AATGATACGGCGACCACCGAGATCTACACCTGCGTGTTATGGTAATTGGCCTACGGGAGGCAGCAG  CAAGCAGAAGACGGCATACGAGATAGCTGCTAAGTCAGTCAGCCGGACTACHVGGGTWTCTAAT |
|  | Endoscope | V3.F.SA505  V4.R.SB703 | AATGATACGGCGACCACCGAGATCTACACTCATCGAGTATGGTAATTGGCCTACGGGAGGCAGCAG  CAAGCAGAAGACGGCATACGAGATAGCTGCTAAGTCAGTCAGCCGGACTACHVGGGTWTCTAAT |
| 30196 | Cloacal | V3.F.SA501  V4.R.SB701 | AATGATACGGCGACCACCGAGATCTACACATCGTACGTATGGTAATTGGCCTACGGGAGGCAGCAG  CAAGCAGAAGACGGCATACGAGATAAGTCGAGAGTCAGTCAGCCGGACTACHVGGGTWTCTAAT |
|  | Endoscope | V3.F.SA502  V4.R.SB701 | AATGATACGGCGACCACCGAGATCTACACACTATCTGTATGGTAATTGGCCTACGGGAGGCAGCAG  CAAGCAGAAGACGGCATACGAGATAAGTCGAGAGTCAGTCAGCCGGACTACHVGGGTWTCTAAT |
|  | Faecal | V3.F.SA503  V4.R.SB701 | AATGATACGGCGACCACCGAGATCTACACTAGCGAGTTATGGTAATTGGCCTACGGGAGGCAGCAG  CAAGCAGAAGACGGCATACGAGATAAGTCGAGAGTCAGTCAGCCGGACTACHVGGGTWTCTAAT |
| 30340 | Endoscope | V3.F.SA501  V4.R.SB704 | AATGATACGGCGACCACCGAGATCTACACATCGTACGTATGGTAATTGGCCTACGGGAGGCAGCAG  CAAGCAGAAGACGGCATACGAGATCATAGAGAAGTCAGTCAGCCGGACTACHVGGGTWTCTAAT |
|  | Cloacal | V3.F.SA502  V4.R.SB704 | AATGATACGGCGACCACCGAGATCTACACACTATCTGTATGGTAATTGGCCTACGGGAGGCAGCAG  CAAGCAGAAGACGGCATACGAGATCATAGAGAAGTCAGTCAGCCGGACTACHVGGGTWTCTAAT |
|  | Faecal | V3.F.SA508  V4.R.SB703 | AATGATACGGCGACCACCGAGATCTACACGACACCGTTATGGTAATTGGCCTACGGGAGGCAGCAG  CAAGCAGAAGACGGCATACGAGATAGCTGCTAAGTCAGTCAGCCGGACTACHVGGGTWTCTAAT |
| 30915 | Endoscope | V3.F.SA501  V4.R.SB711 | AATGATACGGCGACCACCGAGATCTACACATCGTACGTATGGTAATTGGCCTACGGGAGGCAGCAG  CAAGCAGAAGACGGCATACGAGATTCAGCGTTAGTCAGTCAGCCGGACTACHVGGGTWTCTAAT |
|  | Cloacal | V3.F.SA508  V4.R.SB710 | AATGATACGGCGACCACCGAGATCTACACGACACCGTTATGGTAATTGGCCTACGGGAGGCAGCAG  CAAGCAGAAGACGGCATACGAGATTACGAGCAAGTCAGTCAGCCGGACTACHVGGGTWTCTAAT |
| 30943 | Faecal | V3.F.SA501  V4.R.SB709 | AATGATACGGCGACCACCGAGATCTACACATCGTACGTATGGTAATTGGCCTACGGGAGGCAGCAG  CAAGCAGAAGACGGCATACGAGATGTATACGCAGTCAGTCAGCCGGACTACHVGGGTWTCTAAT |
|  | Endoscope | V3.F.SA502  V4.R.SB709 | AATGATACGGCGACCACCGAGATCTACACACTATCTGTATGGTAATTGGCCTACGGGAGGCAGCAG  CAAGCAGAAGACGGCATACGAGATGTATACGCAGTCAGTCAGCCGGACTACHVGGGTWTCTAAT |
|  | Cloacal | V3.F.SA508  V4.R.SB708 | AATGATACGGCGACCACCGAGATCTACACGACACCGTTATGGTAATTGGCCTACGGGAGGCAGCAG  CAAGCAGAAGACGGCATACGAGATGGTACTATAGTCAGTCAGCCGGACTACHVGGGTWTCTAAT |
| 30958 | Endoscope | V3.F.SA504  V4.R.SB712 | AATGATACGGCGACCACCGAGATCTACACCTGCGTGTTATGGTAATTGGCCTACGGGAGGCAGCAG  CAAGCAGAAGACGGCATACGAGATTCGCTACGAGTCAGTCAGCCGGACTACHVGGGTWTCTAAT |
|  | Cloacal | V3.F.SA505  V4.R.SB712 | AATGATACGGCGACCACCGAGATCTACACTCATCGAGTATGGTAATTGGCCTACGGGAGGCAGCAG  CAAGCAGAAGACGGCATACGAGATTCGCTACGAGTCAGTCAGCCGGACTACHVGGGTWTCTAAT |
| 31642 | Cloacal | V3.F.SA502  V4.R.SB712 | AATGATACGGCGACCACCGAGATCTACACACTATCTGTATGGTAATTGGCCTACGGGAGGCAGCAG  CAAGCAGAAGACGGCATACGAGATTCGCTACGAGTCAGTCAGCCGGACTACHVGGGTWTCTAAT |
|  | Endoscope | V3.F.SA503  V4.R.SB712 | AATGATACGGCGACCACCGAGATCTACACTAGCGAGTTATGGTAATTGGCCTACGGGAGGCAGCAG  CAAGCAGAAGACGGCATACGAGATTCGCTACGAGTCAGTCAGCCGGACTACHVGGGTWTCTAAT |
| 32403 | Cloacal | V3.F.SA501  V4.R.SB705 | AATGATACGGCGACCACCGAGATCTACACATCGTACGTATGGTAATTGGCCTACGGGAGGCAGCAG  CAAGCAGAAGACGGCATACGAGATCGTAGATCAGTCAGTCAGCCGGACTACHVGGGTWTCTAAT |
|  | Endoscope | V3.F.SA508  V4.R.SB704 | AATGATACGGCGACCACCGAGATCTACACGACACCGTTATGGTAATTGGCCTACGGGAGGCAGCAG  CAAGCAGAAGACGGCATACGAGATCATAGAGAAGTCAGTCAGCCGGACTACHVGGGTWTCTAAT |
| 32417 | Endoscope | V3.F.SA505  V4.R.SB710 | AATGATACGGCGACCACCGAGATCTACACTCATCGAGTATGGTAATTGGCCTACGGGAGGCAGCAG  CAAGCAGAAGACGGCATACGAGATTACGAGCAAGTCAGTCAGCCGGACTACHVGGGTWTCTAAT |
|  | Cloacal | V3.F.SA506  V4.R.SB710 | AATGATACGGCGACCACCGAGATCTACACCGTGAGTGTATGGTAATTGGCCTACGGGAGGCAGCAG  CAAGCAGAAGACGGCATACGAGATTACGAGCAAGTCAGTCAGCCGGACTACHVGGGTWTCTAAT |
| 32475 | Endoscope | V3.F.SA504  V4.R.SB701 | AATGATACGGCGACCACCGAGATCTACACCTGCGTGTTATGGTAATTGGCCTACGGGAGGCAGCAG  CAAGCAGAAGACGGCATACGAGATAAGTCGAGAGTCAGTCAGCCGGACTACHVGGGTWTCTAAT |
|  | Cloacal | V3.F.SA505  V4.R.SB701 | AATGATACGGCGACCACCGAGATCTACACTCATCGAGTATGGTAATTGGCCTACGGGAGGCAGCAG  CAAGCAGAAGACGGCATACGAGATAAGTCGAGAGTCAGTCAGCCGGACTACHVGGGTWTCTAAT |
| 32620 | Cloacal | V3.F.SA502  V4.R.SB702 | AATGATACGGCGACCACCGAGATCTACACACTATCTGTATGGTAATTGGCCTACGGGAGGCAGCAG  CAAGCAGAAGACGGCATACGAGATATACTTCGAGTCAGTCAGCCGGACTACHVGGGTWTCTAAT |
|  | Endoscope | V3.F.SA503  V4.R.SB702 | AATGATACGGCGACCACCGAGATCTACACTAGCGAGTTATGGTAATTGGCCTACGGGAGGCAGCAG  CAAGCAGAAGACGGCATACGAGATATACTTCGAGTCAGTCAGCCGGACTACHVGGGTWTCTAAT |
| 32625 | Faecal | V3.F.SA502  V4.R.SB710 | AATGATACGGCGACCACCGAGATCTACACACTATCTGTATGGTAATTGGCCTACGGGAGGCAGCAG  CAAGCAGAAGACGGCATACGAGATTACGAGCAAGTCAGTCAGCCGGACTACHVGGGTWTCTAAT |
|  | Cloacal | V3.F.SA503  V4.R.SB710 | AATGATACGGCGACCACCGAGATCTACACTAGCGAGTTATGGTAATTGGCCTACGGGAGGCAGCAG  CAAGCAGAAGACGGCATACGAGATTACGAGCAAGTCAGTCAGCCGGACTACHVGGGTWTCTAAT |
|  | Endoscope | V3.F.SA504  V4.R.SB710 | AATGATACGGCGACCACCGAGATCTACACCTGCGTGTTATGGTAATTGGCCTACGGGAGGCAGCAG  CAAGCAGAAGACGGCATACGAGATTACGAGCAAGTCAGTCAGCCGGACTACHVGGGTWTCTAAT |
| 32627 | Cloacal | V3.F.SA502  V4.R.SB705 | AATGATACGGCGACCACCGAGATCTACACACTATCTGTATGGTAATTGGCCTACGGGAGGCAGCAG  CAAGCAGAAGACGGCATACGAGATCGTAGATCAGTCAGTCAGCCGGACTACHVGGGTWTCTAAT |
|  | Endoscope | V3.F.SA504  V4.R.SB705 | AATGATACGGCGACCACCGAGATCTACACCTGCGTGTTATGGTAATTGGCCTACGGGAGGCAGCAG  CAAGCAGAAGACGGCATACGAGATCGTAGATCAGTCAGTCAGCCGGACTACHVGGGTWTCTAAT |
| 32674 | Endoscope | V3.F.SA506  V4.R.SB703 | AATGATACGGCGACCACCGAGATCTACACCGTGAGTGTATGGTAATTGGCCTACGGGAGGCAGCAG  CAAGCAGAAGACGGCATACGAGATAGCTGCTAAGTCAGTCAGCCGGACTACHVGGGTWTCTAAT |
|  | Cloacal | V3.F.SA507  V4.R.SB703 | AATGATACGGCGACCACCGAGATCTACACGGATATCTTATGGTAATTGGCCTACGGGAGGCAGCAG  CAAGCAGAAGACGGCATACGAGATAGCTGCTAAGTCAGTCAGCCGGACTACHVGGGTWTCTAAT |
| 33105 | Cloacal | V3.F.SA503  V4.R.SB709 | AATGATACGGCGACCACCGAGATCTACACTAGCGAGTTATGGTAATTGGCCTACGGGAGGCAGCAG  CAAGCAGAAGACGGCATACGAGATGTATACGCAGTCAGTCAGCCGGACTACHVGGGTWTCTAAT |
| 33387 | Endoscope | V3.F.SA505  V4.R.SB705 | AATGATACGGCGACCACCGAGATCTACACTCATCGAGTATGGTAATTGGCCTACGGGAGGCAGCAG  CAAGCAGAAGACGGCATACGAGATCGTAGATCAGTCAGTCAGCCGGACTACHVGGGTWTCTAAT |
|  | Cloacal | V3.F.SA506  V4.R.SB705 | AATGATACGGCGACCACCGAGATCTACACCGTGAGTGTATGGTAATTGGCCTACGGGAGGCAGCAG  CAAGCAGAAGACGGCATACGAGATCGTAGATCAGTCAGTCAGCCGGACTACHVGGGTWTCTAAT |
| 33545 | Endoscope | V3.F.SA504  V4.R.SB708 | AATGATACGGCGACCACCGAGATCTACACCTGCGTGTTATGGTAATTGGCCTACGGGAGGCAGCAG  CAAGCAGAAGACGGCATACGAGATGGTACTATAGTCAGTCAGCCGGACTACHVGGGTWTCTAAT |
|  | Cloacal | V3.F.SA505  V4.R.SB708 | AATGATACGGCGACCACCGAGATCTACACTCATCGAGTATGGTAATTGGCCTACGGGAGGCAGCAG  CAAGCAGAAGACGGCATACGAGATGGTACTATAGTCAGTCAGCCGGACTACHVGGGTWTCTAAT |
| 33557 | Cloacal | V3.F.SA504  V4.R.SB706 | AATGATACGGCGACCACCGAGATCTACACCTGCGTGTTATGGTAATTGGCCTACGGGAGGCAGCAG  CAAGCAGAAGACGGCATACGAGATCTCGTTACAGTCAGTCAGCCGGACTACHVGGGTWTCTAAT |
|  | Endoscope | V3.F.SA506  V4.R.SB706 | AATGATACGGCGACCACCGAGATCTACACCGTGAGTGTATGGTAATTGGCCTACGGGAGGCAGCAG |
|  |  |  | CAAGCAGAAGACGGCATACGAGATCTCGTTACAGTCAGTCAGCCGGACTACHVGGGTWTCTAAT |
| 33586 | Cloacal | V3.F.SA501  V4.R.SB706 | AATGATACGGCGACCACCGAGATCTACACATCGTACGTATGGTAATTGGCCTACGGGAGGCAGCAG  CAAGCAGAAGACGGCATACGAGATCTCGTTACAGTCAGTCAGCCGGACTACHVGGGTWTCTAAT |
|  | Faecal | V3.F.SA502  V4.R.SB706 | AATGATACGGCGACCACCGAGATCTACACACTATCTGTATGGTAATTGGCCTACGGGAGGCAGCAG  CAAGCAGAAGACGGCATACGAGATCTCGTTACAGTCAGTCAGCCGGACTACHVGGGTWTCTAAT |
|  | Endoscope | V3.F.SA503  V4.R.SB706 | AATGATACGGCGACCACCGAGATCTACACTAGCGAGTTATGGTAATTGGCCTACGGGAGGCAGCAG  CAAGCAGAAGACGGCATACGAGATCTCGTTACAGTCAGTCAGCCGGACTACHVGGGTWTCTAAT |
| 33743 | Cloacal | V3.F.SA508  V4.R.SB712 | AATGATACGGCGACCACCGAGATCTACACGACACCGTTATGGTAATTGGCCTACGGGAGGCAGCAG  CAAGCAGAAGACGGCATACGAGATTCGCTACGAGTCAGTCAGCCGGACTACHVGGGTWTCTAAT |
| 33747 | Endoscope | V3.F.SA502  V4.R.SB711 | AATGATACGGCGACCACCGAGATCTACACACTATCTGTATGGTAATTGGCCTACGGGAGGCAGCAG  CAAGCAGAAGACGGCATACGAGATTCAGCGTTAGTCAGTCAGCCGGACTACHVGGGTWTCTAAT |
|  | Faecal | V3.F.SA503  V4.R.SB711 | AATGATACGGCGACCACCGAGATCTACACTAGCGAGTTATGGTAATTGGCCTACGGGAGGCAGCAG  CAAGCAGAAGACGGCATACGAGATTCAGCGTTAGTCAGTCAGCCGGACTACHVGGGTWTCTAAT |
|  | Cloacal | V3.F.SA504  V4.R.SB711 | AATGATACGGCGACCACCGAGATCTACACCTGCGTGTTATGGTAATTGGCCTACGGGAGGCAGCAG  CAAGCAGAAGACGGCATACGAGATTCAGCGTTAGTCAGTCAGCCGGACTACHVGGGTWTCTAAT |
| 33929 | Cloacal | V3.F.SA506  V4.R.SB701 | AATGATACGGCGACCACCGAGATCTACACCGTGAGTGTATGGTAATTGGCCTACGGGAGGCAGCAG  CAAGCAGAAGACGGCATACGAGATAAGTCGAGAGTCAGTCAGCCGGACTACHVGGGTWTCTAAT |
|  | Endoscope | V3.F.SA507  V4.R.SB701 | AATGATACGGCGACCACCGAGATCTACACGGATATCTTATGGTAATTGGCCTACGGGAGGCAGCAG  CAAGCAGAAGACGGCATACGAGATAAGTCGAGAGTCAGTCAGCCGGACTACHVGGGTWTCTAAT |
| 34212 | Cloacal | V3.F.SA506  V4.R.SB708 | AATGATACGGCGACCACCGAGATCTACACCGTGAGTGTATGGTAATTGGCCTACGGGAGGCAGCAG  CAAGCAGAAGACGGCATACGAGATGGTACTATAGTCAGTCAGCCGGACTACHVGGGTWTCTAAT |
|  | Endoscope | V3.F.SA507  V4.R.SB708 | AATGATACGGCGACCACCGAGATCTACACGGATATCTTATGGTAATTGGCCTACGGGAGGCAGCAG  CAAGCAGAAGACGGCATACGAGATGGTACTATAGTCAGTCAGCCGGACTACHVGGGTWTCTAAT |
| 34227 | Endoscope | V3.F.SA502  V4.R.SB708 | AATGATACGGCGACCACCGAGATCTACACACTATCTGTATGGTAATTGGCCTACGGGAGGCAGCAG  CAAGCAGAAGACGGCATACGAGATGGTACTATAGTCAGTCAGCCGGACTACHVGGGTWTCTAAT |
|  | Cloacal | V3.F.SA503  V4.R.SB708 | AATGATACGGCGACCACCGAGATCTACACTAGCGAGTTATGGTAATTGGCCTACGGGAGGCAGCAG  CAAGCAGAAGACGGCATACGAGATGGTACTATAGTCAGTCAGCCGGACTACHVGGGTWTCTAAT |
| 39265 | Endoscope | V3.F.SA507  V4.R.SB705 | AATGATACGGCGACCACCGAGATCTACACGGATATCTTATGGTAATTGGCCTACGGGAGGCAGCAG  CAAGCAGAAGACGGCATACGAGATCGTAGATCAGTCAGTCAGCCGGACTACHVGGGTWTCTAAT |
|  | Cloacal | V3.F.SA508  V4.R.SB705 | AATGATACGGCGACCACCGAGATCTACACGACACCGTTATGGTAATTGGCCTACGGGAGGCAGCAG  CAAGCAGAAGACGGCATACGAGATCGTAGATCAGTCAGTCAGCCGGACTACHVGGGTWTCTAAT |
| 39266 | Endoscope | V3.F.SA506  V4.R.SB702 | AATGATACGGCGACCACCGAGATCTACACCGTGAGTGTATGGTAATTGGCCTACGGGAGGCAGCAG  CAAGCAGAAGACGGCATACGAGATATACTTCGAGTCAGTCAGCCGGACTACHVGGGTWTCTAAT |
|  | Cloacal | V3.F.SA507  V4.R.SB702 | AATGATACGGCGACCACCGAGATCTACACGGATATCTTATGGTAATTGGCCTACGGGAGGCAGCAG  CAAGCAGAAGACGGCATACGAGATATACTTCGAGTCAGTCAGCCGGACTACHVGGGTWTCTAAT |
