# Supplementary material for: Helminth burden and ecological factors associated with alterations in wild host gastrointestinal microbiota
Source: ISME J. 2016 Dec 16;11(3):663–75. doi: 10.1038/ismej.2016.153 (PMC5322305; doi:10.1038/ismej.2016.153)
Supplement: Supplementary Table S2 [file ismej2016153x4.docx]

| JN935868 | |
| --- | --- |
| AM259831 | |
| JN976469 | |
| AM259831 | |
| JF218090 | |
| AGWN01000003 | |
| AB849332 | |
| GQ158313 | |
| AJ249327 | |
| AJ276405 | |
| AJ508455 | |
| JX104046 | |
| DQ347943 | |
| JX412111 | |
| AF359531 | |
| GQ867405 | |
| GQ867483 | |
| KC251736 | |
| HM989893 | |
| FM872962 | |
| GQ077190 | |
| DQ793648 | |
| AMCI01001288 | |
| GQ327203 | |
| HM269858 | |
| HM274527 | |
| JQ034227 | |
| FR693359 | |
| HQ231931 | |
| EF093123 | |
| AJ640198 | |
| 27DQ172996 | |
| AB558168 | |
| CP001721 | |
| JN377656 | |
| KM114215 | |
| HQ779009 | |
| AY362901 | |
| EU767500 | |
| X91657 | |
| EU346912 | |
| CU926062 | |
| KJ206760 | |
| DQ917252 | |
| HE818674 | |
| AB594446 | |
| X76566 | |
| KF090700 | |
| AF133537 | |
| AY538692 | |
| AY238506 | |
| FJ459989 | |
| EF187228 | |
| EF621896 | |
| EF621894 | |
| FJ960012 | |
| JN447996 | |
| FJ470493 | |
| JQ470001 | |
| AP012210 | |
| AP012210 | |
| HM124023 | |
| KF681342 | |
| JN713342 | |
| AB680899 | |
| HE583595 | |
| AB681517 | |
| EU873313 | |
| GU593654 | |
| HQ183863 | |
| AJ854484 | |
| AMYT01000015 | |
| DQ113721 | |
| EU772848 | |
| AY188323 | |
| DQ330310 | |
| GU451338 | |
| AJ495802 | |
| GQ259742 | |
| GQ138883 | |
| JPIC01000015 | |
| FM873924 | |
| EF099332 | |
| FJ671784 | |
| DQ352812 | |
| FJ960327 | |
| DQ978216 | |
| DQ234199 | |
| EF445207 | |
| GU227213 | |
| HW066429 | |
| GQ069088 | |
| JF147747 | |
| X84443 | |
| CP007156 | |
| GQ073602 | |
| GQ358800 | |
| GQ358801 | |
| FJ374772 | |
| GQ066289 | |
| CP001779 | |
| HQ397361 | |
| FJ628268 | |
| CP002536 | |
| FR682757 | |
| GU457787 | |
| FM882229 | |
| GQ358807 | |
| HM278045 | |
| HM196766 | |
| AB507489 | |
| HM277729 | |
| GQ358806 | |
| AB211025 | |
| JN713419 | |
| EF071180 | |
| GQ078792 | |
| JJNT01000040 | |
| AOQK01000143 | |
| L34612 | |
| HW066410 | |
| EU289122 | |
| JX871279 | |
| HM318928 | |
| JF201222 | |
| AY794064 | |
| HQ397360 | |
| HQ768619 | |
| DQ456057 | |
| AM710615 | |
| BAHC01000019 | |
| GQ497293 | |
| JN713493 | |
| AY272039 | |
| FJ959814 | |
| FJ959931 | |
| M88138 | |
| AF225549 | |
| AF302108 | |
| HG326498 | |
| HM596295 | |
| HP459625 | |
| FN550117 | |
| JF173870 | |
| AJ549285 | |
| JN033775 | |
| EF516328 | |
| FN550128 | |
| HM265245 | |
| AB286023 | |
| AB286024 | |
| AJ748747 | |
| JN935892 | |
| AY921767 | |
| KP657490 | |
| EU772192 | |
| AY581816 | |
| GQ134177 | |
| AB175732 | |
| JX986976 | |
| GQ175337 | |
| GQ175354 | |
| GQ068015 | |
| KC894531 | |
| CU919748 | |
| JN644765 | |
| GQ358839 | |
| AY771771 | |
| AY772092 | |
| GQ274243 | |
| JX435695 | |
| EF515660 | |
| EU778992 | |
| Z23159 | |
| JN981937 | |
| AB193724 | |
| HG326599 | |
| DQ491453 | |
| DQ307728 | |
| AB522642 | |
| AJ427625 | |
| JQ475779 | |
| X9064 | |
| EU684063 | |
| EU684060 | |
| EU939310 | |
| HE599560 | |
| AY574575 | |
| KF049127 | |
| BD359509 | |
| FM873348 | |
| HM262192 | |
| FJ946553 | |
| HM489989 | |
| HQ171441 | |
| PRJNA19333 | |
| AY654757 | |
| U70715 | |
| U70717 | |
| JQ948045 | |
| KC454305 | |
| JF810495 | |
| AB469787 | |
| FJ366064 | |
| AB187584 | |
| JQ457721 | |
| AY172730 | |
| KF528723 | |
| AF190911 | |
| FJ999734 | |
| AB644260 | |
| X55797 | |
| AWXB01000036 | |
| EU526291 | |
| FJ959656 | |
| GM884215 | |
| FR749776 | |
| JQ407912 | |
| EF559147 | |
| GQ133649 | |
| KJ004406 | |
| KJ004405 | |
| AY048891 | |
| KJ575044 | |
| AB255101 | |
| DQ814600 | |
| KF698726 | |
| AB525416 | |
| JF108289 | |
| JF176541 | |
| JQ425773 | |
| FR693792 | |
| JF776948 | |
| GQ358820 | |
| AB255084 | |
| JQ975901 | |
| JQ337096 | |
| JX530073 | |
| JX559230 | |
| KC000981 | |
| HF569157 | |
| AB681720 | |
| JF800153 | |
| AM403661 | |
| HM308241 | |
| JF179545 | |
| JN790962 | |
| FJ195995 | |
| KF441621 | |
| AF409012 | |
| KC454317 | |
| HQ286278 | |
| HQ286279 | |
| HQ286282 | |
| EU471774 | |
| FJ960055 | |
| HE681248 | |
| AY167339 | |
| M96746 | |
| DQ167249 | |
| HM326644 | |
| JF116525 | |
| EU777117 | |
| AY949857 | |
| KJ783119 | |
| DQ129575 | |
| AY744399 | |
| AF001637 | |
| DQ186925 | |
| X52172 | |
| U20797 | |
| U65908 | |
| AF001653 | |
| AF001650 | |
| EF182722 | |
| EU276571 | |
| U90216 | |
| AB681542 | |
| KJ606800 | |
| HM262179 | |
| FR691451 | |
| GU994083 | |
| FR749726 | |
| KF188473 | |
| EF061907 | |
| JN713542 | |
| HG421076 | |
| KF656772 | |
| JQ582693 | |
| JX948589 | |
| JX948654 | |
| AY936189 | |
| DQ071142 | |
| AJ832129 | |
| NR044681 | |
| NR042279 | |
| NC_008319 | |
| EU475436 | |
| KC884005 | |
| CU925597 | |
| FJ671878 | |
| M34115 | |
| GQ136200 | |
| EU009749 | |
| HE576081 | |
| GQ135821 | |
| FR682696 | |
| JQ083944 | |
| DQ798020 |  |
| FJ959947 |  |
| FJ960123 |  |
| JF230013 |  |
| EF108444 |  |
| AB286539 |  |
| EU137432 |  |
| HQ758155 |  |
| HQ805210 |  |
| JQ463442 |  |
| AUAN01000035 |  |
| AB639142 |  |
| AF186071 |  |

**Table S2** Accession numbers of published sequences from other studies used in the creation of the phylogenetic tree.
